# Three-dimensional network of filamentary currents and super-thermal electrons during magnetotail magnetic reconnection

**DOI:** 10.1038/s41467-022-31025-9

**Published:** 2022-06-10

**Authors:** Xinmin Li, Rongsheng Wang, Quanming Lu, Christopher T. Russell, San Lu, Ian J. Cohen, R. E. Ergun, Shui Wang

**Affiliations:** 1 CAS Key Laboratory of Geospace Environment, Department of Geophysics and Planetary Science, University of Science and Technology of China, Hefei, 230026 China; 2 CAS Center for Excellence in Comparative Planetology, Hefei, China; 3 Anhui Mengcheng Geophysics National Observation and Research Station, University of Science and Technology of China, Mengcheng, 233500 Anhui, China; 4 Earth Planetary and Space Sciences, University of California, Los Angeles, CA 90095 USA; 5 The Johns Hopkins University Applied Physics Laboratory, Laurel, MD USA; 6 Department for Astrophysical and Planetary Sciences, University of Colorado, Boulder, CO USA

**Keywords:** Magnetospheric physics, Magnetospheric physics

## Abstract

Magnetic reconnection is a fundamental plasma process by which magnetic field lines on two sides of the current sheet flow inward to yield an X-line topology. It is responsible for producing energetic electrons in explosive phenomena in space, astrophysical, and laboratorial plasmas. The X-line region is supposed to be the important place for generating energetic electrons. However, how these energetic electrons are generated in such a limited region is still poorly understood. Here, using Magnetospheric multiscale mission data acquired in Earth’s magnetotail, we present direct evidence of super-thermal electrons up to 300 keV inside an X-line region, and the electrons display a power-law spectrum with an index of about 8.0. Concurrently, three-dimensional network of dynamic filamentary currents in electron scale is observed and leads to electromagnetic turbulence therein. The observations indicate that the electrons are effectively accelerated while the X-line region evolves into turbulence with a complex filamentary current network.

## Abstract (web summary)

Magnetotail reconnection plays an important role in explosive energy conversion. Here, the authors show direct evidence of super-thermal electrons up to 300 keV within X-line region in Earth’s magnetotail, indicating effective electron acceleration due to turbulence.

## Introduction

Magnetic reconnection can explosively convert magnetic free energy into plasma kinetic energy and heating^1–6^, and accounts for a large number of explosive phenomena in nature^1,7^. Plasma turbulence is frequently detected in the process of magnetic reconnection^8–11^ and is generally associated with the high-speed reconnection ion outflows^12–14^. The turbulence in reconnection is commonly attributed to bursty outflows and can facilitate energy dissipation^9,15^ and energize electrons to form the power-law distribution^16^.

Recent 3-D simulations suggested that the electron diffusion region (EDR) would disintegrate into a complex web of filamentary currents (FCs) leading to the development of turbulence^17^. The electron Kelvin-Helmholtz instability (EKHI)^18^ and the lower hybrid drift instability (LHDI)^10,19^ can be responsible for the generation of the turbulence at the X-line region. In the process of the EKHI, a series of magnetic vortices would expand rapidly, and thus a strong electric field was induced inside them^18^. The induced electric field can efficiently accelerate the electrons to form a power-law spectrum. The power-law index is determined by the ratio of the spatial scale of the inductive electric field and that of vortices^18^. The FCs are detected in the reconnection outflow^20^ and also inside magnetic flux ropes^21–23^. What roles of these FCs play in the reconnection remain elusive.

In this work, using the Magnetospheric Multiscale (MMS) mission^24^ measurement in the magnetotail, we establish the relation between FCs and turbulence inside the X-line region. We find that the electrons can be effectively accelerated to relativistic energy due to turbulence.

## Results

### Overview of the reconnection event

**Fig. 1:**
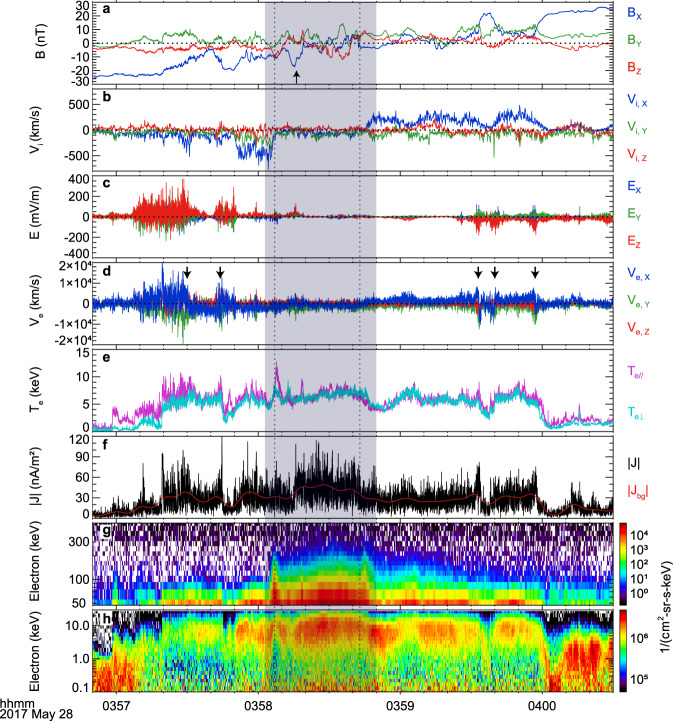
Overview of the turbulent magnetic reconnection. **a** Three components of the magnetic field. The black arrow marks the temporary excursion (03:58:10–03:58:25 UT) when the spacecraft shortly left the current sheet center and then returned again. **b** The ion bulk flows. **c** Three components of the electric field. **d** The electron bulk flows. The black arrows represent the separatrix regions. **e** Parallel and perpendicular electron temperatures. **f** The magnitude of current density (black curve), and the background current density (red curve). **g** Energetic electron (47–500 KeV) omnidirectional differential flux. **h** Electron (0.1–30 KeV) omnidirectional differential flux. The shadow area corresponds to the presence of energetic electrons. The area between two vertical black dashed lines at 03:58:07 and 03:58:43 UT represents the X-line region.

An intense substorm activity persisted for about 20 h from 22:00 UT on 27 May 2017. During this time interval, all four MMS satellites were located in the magnetotail plasma sheet (See Supplementary Fig. 1) and detected several events of ion flow reversal. Here, we concentrate on the event at around 4:00 UT on 28 May when MMS was at [−19.2, −11.3, 3.2] Re in Geocentric Solar Ecliptic (GSE) coordinates. In this event, the Alfvénic ion flow reversed from tailward to earthward and then continued for more than one hour (See Supplementary Fig. 2). In this work, we will concentrate on the time interval of the ion flow reversal shown in Fig. 1. The data from MMS 1 was used unless otherwise stated.

The ion flow reversed at around 03:58:45 UT, from tailward to earthward (Fig. 1b) as the spacecraft crossed the plasma sheet from the southern hemisphere (${B}_{x} < 0$) to the northern hemisphere (${B}_{x} > 0$, Fig. 1a). The tailward ion flow ${v}_{ix}$ was down to −700 km/s (0.4 **V**_*A*_) at around 03:58:04 UT, and the earthward flows exceeded 400 km/s (0.24 **V**_*A*_) at around 03:59:50 UT, where **V**_*A*_ is 1700 km/s, based on *N* = 0.1 cm^−3^ and |**B**| = 25 nT. It indicates that MMS passed through an X-line region from tailward to earthward. The electron bulk flow ${v}_{ex}$ (blue trace in Fig. 1d) was much stronger than the ion flow and displayed a similar overall reversal at around 03:58:45 UT. Moreover, ${v}_{ex}$ showed a few more reversals with simultaneous enhancements of electric field fluctuations (Fig. 1c), e.g., at around 03:57:30, 03:57:44, 03:59:33, 03:59:40, and 03:59:57 UT (the arrows at the top of Fig. 1d), which could correspond to the separatrix regions. The electric field *E*_*z*_ was mainly positive below the current sheet and negative above it (red trace in Fig. 1c), consistent with the Hall electric field. The electrons were significantly heated to 8 keV during the ion flow reversal (Fig. 1e, h). Based on the analysis above, it is concluded that the spacecraft entered the ion diffusion region during this time interval.

The ion flow ${v}_{ix}$ did not reverse gradually, as reported previously^25–27^. In contrast, it decreased steeply from −700 to −100 km/s within 3 s (03:58:04 −03:58:07 UT), then kept a low value (<100 km/s) for 36 s (03:58:07–03:58:43 UT, i.e., the X-line region), and finally increased sharply to ~350 km/s. In the X-line region, |*B*_*x*_| was <20 nT and changed the sign several times. It indicates that MMS was basically located around the current sheet center. According to the standard collisionless reconnection model^28–31^ and previous observations^25–27^, an electron current layer with a thickness of a few electron inertial lengths (*d*_*e*_) should have been detected. The fact is that many current spikes were detected instead of a single compact electron current layer (Fig. 1f). The current density was very strong, sometimes over 100 nA/m^2^, inside the X-line region.

### Three-dimensional network of FCs in the X-line region

**Fig. 2:**
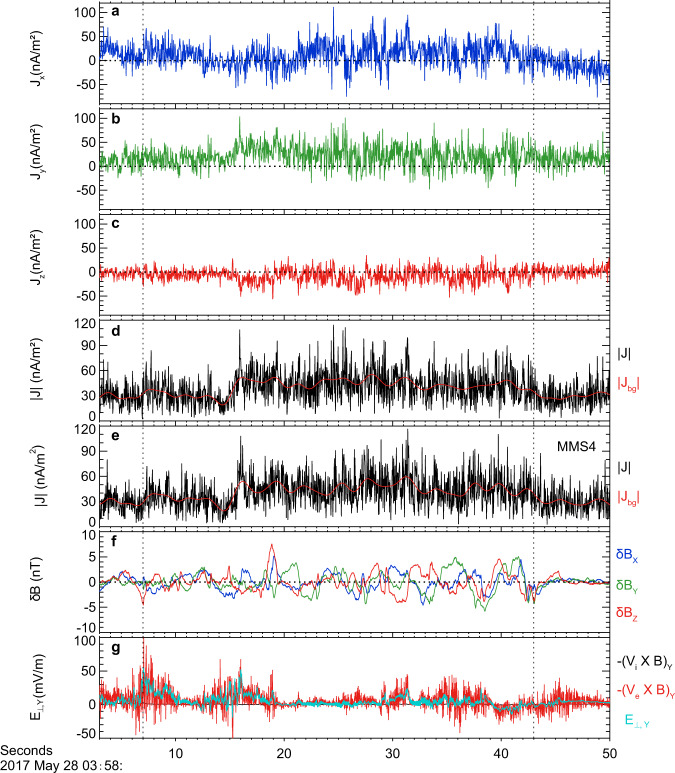
Features of the filamentary currents around the X-line. **a**–**c** Three components of the current density. **d** The magnitude of current density (black curve), and the background current density (red curve) observed by MMS1. **e** The magnitude of current density (black curve), and the background current density (red curve) observed by MMS4. **f** Three components of the disturbed magnetic field *δB*. The background magnetic fields below ***f***_***ci***_ (0.14 Hz) have been removed. **g** The comparisons between **E**_⊥_ with $-({{{{{{\bf{V}}}}}}}_{e}\times {{{{{\bf{B}}}}}})$ and $-({{{{{{\bf{V}}}}}}}_{{{{{{\rm{i}}}}}}}\times {{{{{\bf{B}}}}}})$ in **Y**-GSE direction. The area between two vertical black dashed lines at 03:58:07 and 03:58:43 UT represents the X-line region.

**Fig. 3:**
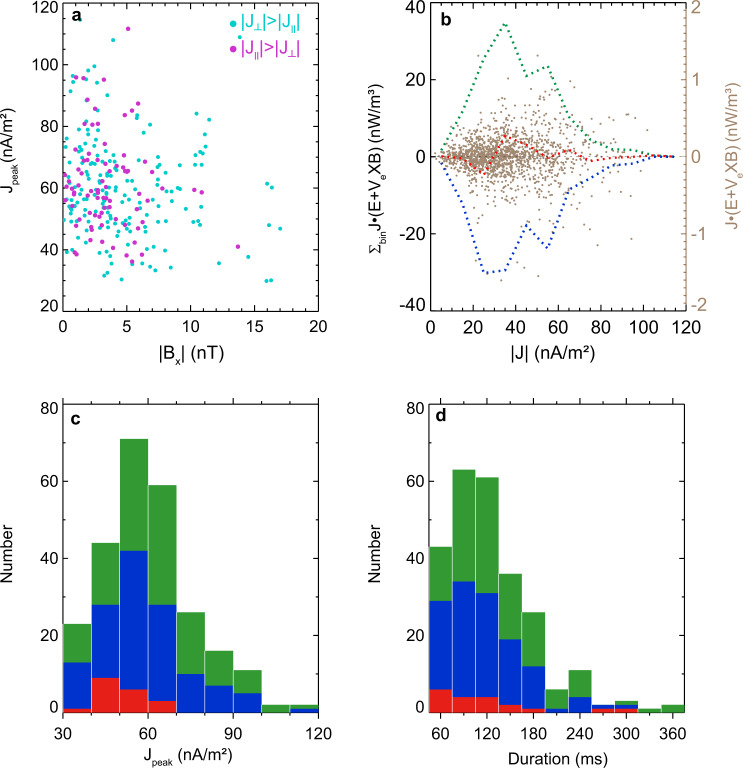
Average features of current spikes. **a** The scatter plot of *B*_*x*_ and the peak values of the current spikes ${J}_{{{{{\rm{peak}}}}}}$. The blue and pink dots represent the spikes dominated by the perpendicular and parallel currents, respectively. **b** The scatter plot of ${{{{{\bf{J}}}}}}\cdot ({{{{{\bf{E}}}}}}{{{{{\boldsymbol{+}}}}}}{{{{{{\bf{V}}}}}}}_{{{{{{\bf{e}}}}}}}\times {{{{{\bf{B}}}}}})$ and current density intensity |*J*|. The left axis shows the sum of ${{{{{\bf{J}}}}}}\cdot ({{{{{\bf{E}}}}}}+{{{{{{\bf{V}}}}}}}_{{{{{{\bf{e}}}}}}}\times {{{{{\bf{B}}}}}})$ in each current bin. The green and blue dashed lines are the sums of positive and negative ${{{{{\bf{J}}}}}}\cdot ({{{{{\bf{E}}}}}}+{{{{{{\bf{V}}}}}}}_{{{{{{\bf{e}}}}}}}\times {{{{{\bf{B}}}}}})$ in each current bin, and the red dashed line is the sum of ${{{{{\bf{J}}}}}}\cdot ({{{{{\bf{E}}}}}}+{{{{{{\bf{V}}}}}}}_{{{{{{\bf{e}}}}}}}\times {{{{{\bf{B}}}}}})$ (net dissipation) in each current bin. The bin size is 10 nA**/**m^2^. **c**, **d** The histogram of the number and duration of the current spikes. The blue, green, and red bars represent the currents of spikes dominated in the *x*, *y*, and *z* components, respectively. The data used in Fig. 3 are from the time period (03:58:07–03:58:43 UT) marked by two vertical black dashed lines when the MMS was located in the X-line region.

Figure 2a–d shows three components and the magnitude of the current density around the X-line region. *J*_*x*_ and *J*_*y*_ were stronger than *J*_*z*_. *J*_*y*_ was primarily positive. The currents in all three directions (Fig. 2a–c) displayed well-separated spikes, and so did the total current density (Fig. 2d). It means that the current sheet had been fully fragmented. In order to determine the common features of these current spikes, we identified all of the spikes with a local maximum >30 nA/m^2^ in Fig. 2d (See “Methods, Identification of current spikes”). A total of 254 current spikes were identified inside the X-line region. The relation between the peak values of the |**J**| spikes and |*B*_*x*_| can be found in Fig. 3a. Overall, the peak values declined as |*B*_*x*_| became large, in agreement with the Harris-type current sheet in the magnetotail^26,27,30,32^. The spikes dominated by the parallel current (pink points) were concentrated closer to the center (*B*_*x*_ = 0) than those dominated by the perpendicular component (blue points), consistent with the current profile across EDR^27^. Thus, it seems that the current spikes were caused by the fragmentation of the whole compact electron current layer, namely EDR.

These spikes were classified into nine groups according to their intensities from 30 to 120 nA/m^2^. Figure 3c displays the number of spikes versus the current intensity and the blue, green, and red bars represent the currents dominated in *x*, *y*, and *z* components, respectively. The spikes with a current below 70 nA/m^2^ occupied about 80%. For the intense current above 70 nA/m^2^, it was dominated in *y* or *x* components while all three components could dominate in the spikes with a current below 70 nA/m^2^. Because the current needs to be closed, these current spikes must be intertwined and thereby generate a three-dimensional current network.

Inside the X-line region, there was a temporary excursion (03:58:10–03:58:25 UT) with a *B*_*x*_ minimum of −18 nT (marked by the black arrow in Fig. 1a). It indicates that the spacecraft shortly left the background current sheet center and then returned. Thus, the speed of the plasma sheet relative to the spacecraft was roughly estimated to be about 250 km/s (See “Methods, Estimation of the current sheet speed”). The duration of the spikes was very short (Fig. 3d) and <180 ms for most of them (90%). Given the various durations between 360 and 90 ms, their thicknesses varied from 5.4 and 1.3 *d*_*e*_, where *d*_*e*_ = 17 km is the electron inertial length based on *N* = 0.1 cm^−3^. These current spikes corresponded to FCs. Therefore, a complex three-dimensional FC system was observed in the X-line region, analogous to the web of current filaments in numerical simulations^17^.

**Fig. 4:**
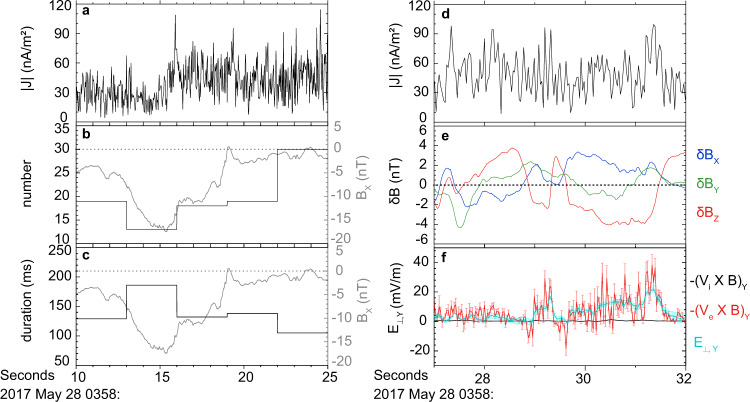
The spatial variation of the filamentary current and its correspondence to a disturbed magnetic field. **a** The magnitude of current density during 03:58:10–3:58:25 UT, when the spacecraft shortly left the current sheet center and then returned again. **b** The number of the current spikes within every three seconds. **c** The average duration of the current spikes within every three seconds. **d** The magnitude of current density during 03:58:27–03:58:32 UT. **e** Three components of the disturbed magnetic field *δB*. The background magnetic fields below ***f***_***ci***_ (0.14 Hz) have been removed. **f** The comparisons between **E**_⊥_ with $-({{{{{{\bf{V}}}}}}}_{e}\times {{{{{\bf{B}}}}}})$ and $-({{{{{{\bf{V}}}}}}}_{{{{{{\rm{i}}}}}}}\times {{{{{\bf{B}}}}}})$ in Y-GSE direction with error bars showing the measurement errors.

Due to the temporary excursion, the spatial distribution of the FCs can be explored in detail. The currents during this excursion are enlarged in Fig. 4a–c. The number of the FCs within every three seconds was anti-correlated to |*B*_*x*_| (Fig. 4b). Assuming the plasma sheet was moving with a constant speed during this short interval, it indicates that more and more FCs were detected as the spacecraft approached the center. Furthermore, the positive correlation between the average duration of the FCs and |*B*_*x*_| (Fig. 4c) suggests that FCs became increasingly thinner as the spacecraft approached the center. The separation of MMS4 and MMS1 was only 3.2 *d*_*e*_ (See Supplementary Fig. 1). The current spikes were dramatically different between them (Fig. 2d, e), although the background current profile (the red curves) was similar. It indicates the FCs were not only thin but also dynamically active, as observed previously inside flux ropes^21,22^.

Because of thin and dynamic FCs in the X-line region, electric field and magnetic field fluctuations were very strong (Fig. 2f, g). The variation of the magnetic field *δ****B*** and the electric field was intense inside the X-line region. *δ****B*** was related to the FCs (Fig. 2d, f). The intense disturbed magnetic field was mainly observed at the region with strong current spikes. It indicates that the thin and dynamic current filaments can generate strong magnetic field fluctuations. This relation can be found more clearly in Fig. 4d, e, where FCs in a short interval near *B*_*x*_ = 0 were expanded. In Fig. 4f, the perpendicular electric field ${E}_{\perp ,y}$, $-{({{{{{{\bf{V}}}}}}}_{{{{{{\bf{e}}}}}}}\times {{{{{\bf{B}}}}}})}_{y}$, $-{({{{{{{\bf{V}}}}}}}_{i}\times {{{{{\bf{B}}}}}})}_{y}$ are displayed. $-{({{{{{{\bf{V}}}}}}}_{i}\times {{{{{\bf{B}}}}}})}_{y}$ was close to zero and distinct from $-{({{{{{{\bf{V}}}}}}}_{{{{{{\bf{e}}}}}}}\times {{{{{\bf{B}}}}}})}_{y}$ and ${E}_{\perp ,y}$. Moreover, the difference between $-{({{{{{{\bf{V}}}}}}}_{{{{{{\bf{e}}}}}}}\times {{{{{\bf{B}}}}}})}_{y}$ and ${E}_{\perp ,y}$ was evident. It means that the ions and electrons were both decoupled from the magnetic field lines inside these FCs. This situation was the same also inside the whole X-line region (Fig. 2g).

Since the electrons were decoupled from magnetic field lines, the non-ideal electric field would be generated. Considering the difference between $-{({{{{{{\bf{V}}}}}}}_{{{{{{\bf{e}}}}}}}\times {{{{{\bf{B}}}}}})}_{y}$ and ${E}_{\perp ,y}$ varied largely (Fig. 2g), the non-ideal electric field should be developed non-uniformly in the X-line region. The energy dissipation in the electron frame^33^ ${{{{{\bf{J}}}}}}\cdot ({{{{{\bf{E}}}}}}+{{{{{{\bf{V}}}}}}}_{e}\times {{{{{\bf{B}}}}}})$ was intense but randomly negative or positive. The negative and positive values were separately summed in each current bin and the results were shown in blue and green dotted curves in Fig. 3b, respectively. The net ${{{{{\bf{J}}}}}}\cdot ({{{{{\bf{E}}}}}}+{{{{{{\bf{V}}}}}}}_{e}\times {{{{{\bf{B}}}}}})$ within each bin was shown in red. The energy dissipation strongly depended on the intensity of the current density. In the region with weak current (<30 nA/m^2^), the negative ${{{{{\bf{J}}}}}}\cdot ({{{{{\bf{E}}}}}}+{{{{{{\bf{V}}}}}}}_{e}\times {{{{{\bf{B}}}}}})$ means a dynamo process there. In the region with a current larger than 30 nA/m^2^, ${{{{{\bf{J}}}}}}\cdot ({{{{{\bf{E}}}}}}+{{{{{{\bf{V}}}}}}}_{e}\times {{{{{\bf{B}}}}}})$ was basically positive. Namely, magnetic energy was released to energize plasma.

**Fig. 5:**
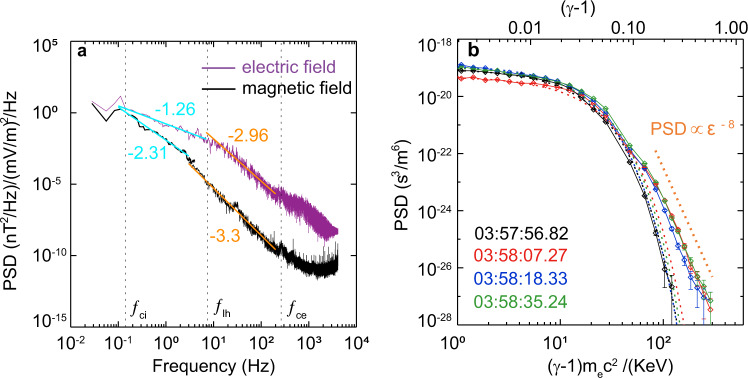
Power spectral density of electromagnetic fields and electron distribution functions. **a** Power spectral density of the ${E}_{x}+{E}_{y}$(purple curve) and the *B*_*z*_(black curve) during 03:58:07–03:58:43 UT (X-line region). The vertical dashed lines represent the average ion cyclotron frequency (*f*_*ci*_ = 0.14 Hz), average lower-hybrid frequency (*f*_*lh*_ = 7 Hz), and average electron cyclotron frequency (*f*_*ce*_ = 260 Hz). The colored lines are power-law fits to specific frequency bands. **b** Electron distribution functions at different times (the black trace represents the background) with error bars showing the uncertainty as $1/\sqrt{N}$, where *N* is the total number of counts in each energy channel. The dashed lines show two pieces of fitting, the Maxwell fitting, and the power-law fitting. Data Points with >100% uncertainty have been removed. The top axis represents the relativistic factor.

Figure 5a shows the power spectral density (PSD) of *B*_*z*_ (black curve) and ${E}_{y}+{E}_{x}$ (purple curve) inside the X-line region. The PSD of magnetic and electric fields both followed the power laws and had a spectral break near the lower-hybrid frequency (*f*_*lh*_). Between ion cyclotron frequency (*f*_*ci*_) and lower-hybrid frequency (*f*_*lh*_), the spectral index of the magnetic field was −2.31, while the electric field had a shallow spectral index (−1.26). Above *f*_*lh*_, magnetic and electric fields had steeper spectra, and their indexes were −3.3 and −2.96, respectively. It indicates that the diffusion region had evolved into a turbulent state while MMS crossed it. This turbulence could be generated by the thin and dynamic current network. The FCs were also detected outside of the X-line region (before 03:58:07 UT and after 03:58:43 UT) and were concurrent with the increases in the ion flow ${v}_{ix}$ (Fig. 1b, f). The generation of these FCs can be due to the reconnection outflows as reported previously^12–14,20^, different from the FCs investigated in this work, and will be studied in future work.

### Electron acceleration inside the turbulent X-line region

Figure 1g and h shows the differential fluxes of electrons in the energy ranges of 47–500 keV and 0.1–30 keV, respectively. The electron fluxes were greatly heightened from 2 to about 300 keV in the vicinity of the X-line region (the shadow area in Fig. 1). The fluxes of electrons above 50 keV increased by two orders of magnitude relative to the background value. This indicates that the electrons were substantially energized up to 300 keV in the X-line region, relative to the thermal electrons <1 keV at around 03:57 UT. The energetic electrons displayed a power-law distribution with a nearly consistent index of 8.0 in the X-line region (Fig. 5b).

**Fig. 6:**
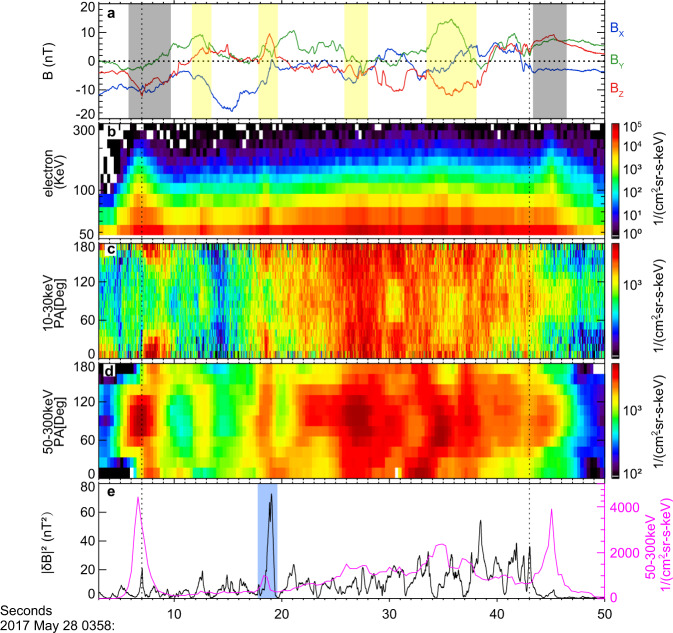
Observation of electron acceleration around the X-line region. **a** Three components of the magnetic field. The yellow and gray bars represent the further localized enhancements of energetic electron fluxes. **b** Energetic electron (47–300 KeV) omnidirectional differential flux. **c** Electron pitch angle distribution during 10–30 KeV. **d** Electron pitch angle distribution during 50–300 KeV; **e** The total disturbed magnetic energy below the ions scale (the frequency great than ***f***_***ci***_), |*δ****B***|^2^, and the total flux during 30–300 KeV (pink curve). The blue bar corresponds to an isolated 3D vortex structure. The area between two vertical black dashed lines at 03:58:07 and 03:58:43 UT represents the X-line region.

Although the energetic electron fluxes maintained a high level in the X-line region, a few further localized enhancements were detected (Fig. 6b) and corresponded to the gray and yellow bars in Fig. 6a. The further enhancements at around 03:58:06 and 03:58:43 UT were clear and correlated to the strong |*B*_*Z*_| (Fig. 6a) at the two ends of the X-line region. At the tailward end (the first bar) with ${{{{{{\rm{B}}}}}}}_{{{{{{\rm{Z}}}}}}} < 0$, the flux enhancement first appeared at around 90° from 50 to 300 keV at 03:58:04 UT, and 3 s later, the enhancements began to occur at 0° and 180° also from 10 to 300 keV (Fig. 6c, d). At the earthward end with ${{{{{{\rm{B}}}}}}}_{{{{{{\rm{Z}}}}}}} > 0$ (03:58:43 UT, the last bar), the flux enhancement was only observed at 90°. Immediately out of the two ends, the ion bulk flow ${v}_{ix}$ was sharply intensified (Fig. 1b). Thus, the two ends with strong |*B*_*Z*_| corresponded to the pile-up regions of the magnetic field *B*_*Z*_. At the pile-up regions, the electrons would be accelerated in the perpendicular direction since the gradient drift was along the induced electric field direction, as suggested in simulations^34^.

Inside the X-line region, further flux enhancements were observed at least at four places (around 03:58:13, 03:58:19, 03:58:27, and 03:58:35 UT, yellow bars in Fig. 6a). At around 03:58:19 and 03:58:35 UT, the further enhancements were associated with a peak and a valley of *B*_*Z*_, respectively. The electrons displayed field-aligned bi-directional distribution at energies of 10–30 keV and 90° flux increase at energies of 50–300 keV at these two places (Fig. 6c, d). There was another deep valley of *B*_*Z*_ from 03:58:29 to 03:58:32 UT, and energetic electron fluxes were moderately enhanced, with a similar pitch angle distribution to those at the *B*_*Z*_ peak and valley mentioned above. Since the similar pitch angle distribution of energetic electrons was observed, the acceleration mechanisms could be the same at those locations and be related to some kinds of magnetic structures. The field-aligned bi-directional distribution of the electrons at the relatively low energy (10–30 keV) indicates these magnetic structures could be closed. The betatron acceleration^34^ could be responsible for the flux enhancements at the 90° direction.

Another two further flux enhancements were observed at around 03:58:13 and 03:58:27 UT when no clear peak or valley of *B*_*Z*_ was detected. The energetic electrons from 10 to 300 keV exhibited flux enhancements at 90°, and the field-aligned bi-directional distribution disappeared. The 90° flux enhancement for the electrons above 50 keV (Fig. 6d) was persistently observed from 03:58:21 to 03:58:46 UT. Namely, this kind of distribution was common for the electrons above 50 keV inside the turbulent diffusion region. In contrast, the field-aligned bi-directional distribution was primarily associated with the peak or valley of *B*_*Z*_. In addition, the flux enhancement merely at 0° can be occasionally observed, e.g., at around 03:58:20, 03:58:33 UT in Fig. 6d. The complex electron pitch angle distribution indicates the electrons could experience multiple acceleration mechanisms inside the turbulent diffusion region.

The fluxes of energetic electrons at energies of 50–300 keV were collocated in Fig. 6e with the disturbed magnetic field energy density (|*δ***B**|^2^). |*δ***B**|^2^ had much more peaks than the fluxes. In addition to the isolated peaks at the pile-up regions, the electron fluxes kept a relatively high level between 03:58:18 and 03:58:44 UT when amplitudes of |*δ***B**|^2^ peaks were large too. The correlation between fluxes and the disturbed magnetic field energy density indicates that electron acceleration inside the X-line region was related to turbulence. Given the special magnetic structures inside the turbulent diffusion region, the electrons would experience second-order Fermi acceleration^35^ to gain energy. The complex magnetic topology inside the turbulent diffusion region would extend the electron dwelling time^36,37^ and thus allow the electrons to be persistently accelerated by the non-ideal electric field to form the power-law distribution. The field-aligned flux enhancements indicate that the parallel electrostatic potential^38^ could also contribute to the electron acceleration.

## Discussion

**Fig. 7:**
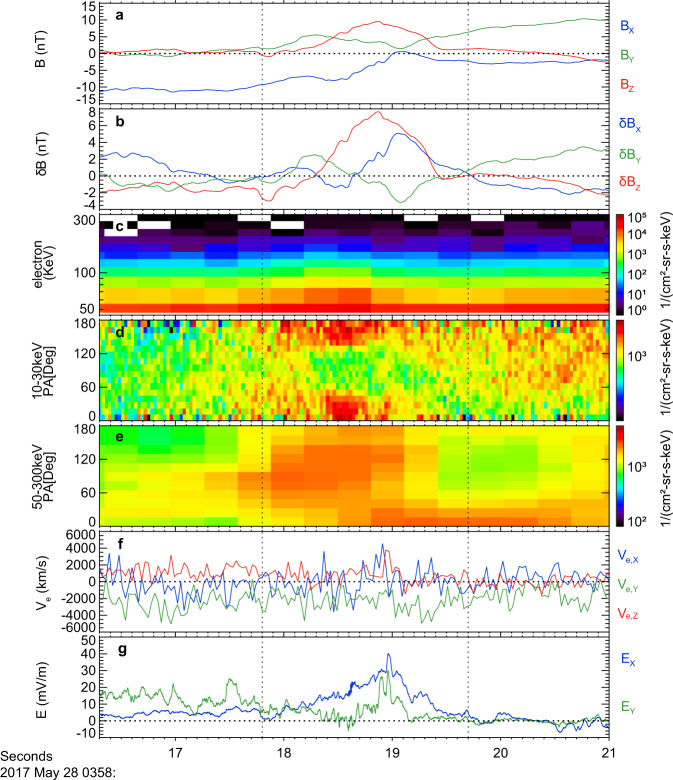
The observation of a potential vortex structure. **a** Three components of the magnetic field. **b** Three components of the disturbed magnetic field *δ****B***. **c** Energetic electron (47–300 KeV) omnidirectional differential flux**. d** Electron pitch angle distribution during 10–30 KeV. **e** Electron pitch angle distribution during 50–300 KeV; **f** The electron bulk flows; **g** Two components of the electric field, *E*_*x*_ and *E*_*y*_.

Inside the turbulent diffusion region, it is hard to determine exact electron acceleration mechanisms, because electrons might undergo a few acceleration processes. Recent simulations show that the electrons can be efficiently accelerated to form a power-law by the induced electric field inside magnetic vortices due to EKHI^18,39^, namely second-order Fermi acceleration. The index varied from 7 to 3 as EKHI evolved from a linear phase to a non-linear phase. In the event investigated in this study, we find some special magnetic structures inside the diffusion region, which could be the magnetic vortices resulting from EKHI. One of the structures at around 03:58:19 UT, marked by a blue bar in Fig. 6e and expanded in Fig. 7, was like the magnetic vortex in the EKHI simulation.

A clear *B*_*z*_ peak was preceded by a slightly negative *B*_*z*_ (left vertical dashed line) in Fig. 7a. Such asymmetric bipolar *B*_*z*_ with a small *B*_*y*_ peak could be a magnetic vortex, i.e., magnetic flux rope. It became more apparent in *δ***B** (Fig. 7b). Within the structure, the strong induced electric field (Fig. 7g and “Methods, Estimation of the induced electric field”), and the energetic electrons up to 100 keV (Fig. 7c) with the power-law distributions (blue trace in Fig. 5b) were detected. The angle distribution shows that the 90° flux enhancement was observed at energy above 50 keV (Fig. 7e) while the 0° and 180° enhancements were found in the lower energy of 10–30 keV (Fig. 7d). Our observations confirmed the electron acceleration inside the magnetic vortex, which is consistent with the EKHI simulations^18,39^. However, no significant electron shear flow was detected inside the magnetic vortex (Fig. 7f). Considering the asymmetric bipolar *B*_*z*_, the spacecraft should have missed the cross-section center of the flux rope and thus cannot detect the electron flow vortex. The weak shear flow in the z-direction could be part of the electron vortex inside the structure.

The observations indicate that EKHI could be important for electron acceleration inside the X-line region. However, some remarkable differences between our observation and the simulation exist. In our event, a complex magnetic topology was observed in the X-line region, and only one potential magnetic vortex was discerned, distinct from the simulations where a series of regular magnetic vortices were produced. More importantly, the index in the observed turbulent diffusion region was about 8, much softer than that in simulations where the index became harder from 7.5 in the initial linear stage to 3.5 at the non-linear stage. The initial current sheet structure, plasma parameter, and guide field intensity were also quite different between the observation and simulations. The current sheet generally tends to be Harris-type with a weak guide field in the magnetotail, while a force-free current sheet with a strong guide field was adopted in simulations. The plasma beta and the ratio of electron plasma frequency to electron cyclotron frequency were much larger in observation than those in simulations. Furthermore, the simulations are two-dimensional, while our observations are a three-dimensional process that would allow the growth of other instabilities^18^. Given so many dramatic differences between observations and simulations, more efforts are needed to evaluate the role of EKHI in electron acceleration during reconnection and whether EKHI can really occur in the regime of the magnetotail reconnection, even though a potential magnetic vortex was detected in the observation.

Another issue is how these FCs are formed inside the X-line region. During magnetic reconnection, a laminar electron current layer should be generated at first^26,27^ and then is repeatedly fragmented into a lot of small electron current filaments, resulting in the FC network. The EKHI can be one potential candidate^18,39^. The oblique tearing mode instability^40^, the waves in the lower-hybrid frequency range^19^, and the whistler-mode waves^17^ can be other possibilities for the generation of the FC network. Since the waves were very rich inside the X-line region, e.g., whistler-mode waves, lower-hybrid waves, and high-frequency electrostatic waves (See Supplementary Figs. 3 and 4). Further studies are needed to resolve this issue.

In summary, a three-dimensional network of FCs was observed in the X-line region of turbulent magnetic reconnection and was the reason for the generation of the turbulence. Due to the turbulence in the X-line region, the electrons can be continuously accelerated by the turbulent electric field to form a power-law spectrum.

## Methods

### Instruments and database

The data from several instruments onboard MMS are used. The magnetic field and electric field data are respectively measured by the Flux Gate Magnetometer (FGM)^41^ sampling at 128/s and electric field double probe (EDP)^42,43^ sampling at 8192/s. The plasma data are obtained from the Fast Plasma Investigation (FPI)^44^. The time resolution is 30 ms for electrons and 150 ms for ions. The energetic electrons data are taken from the Fly’s Energetic Particle Spectrometer (FEEPS)^45,46^ with a 300 ms time resolution. The magnetic field fluctuation data are provided by the tri-axial search-coil magnetometer (SCM)^47^ with a time resolution of 8192 in burst mode.

### Estimation of the current sheet speed

The speed of the current sheet was obtained from a multi-spacecraft method (timing method)^48^. The differences in the position of the four MMS satellites will cause the time delay when satellites pass through the current sheet. The relative position of the four MMS satellites is known. Thus, the speed of the current sheet can be obtained. The timing method was performed to the points of *B*_*x*_ = −13 nT during 03:58:15–03:58:19 UT. The results showed that the speed of the current sheet was about 250 km/s.

### Higher-order data products

Current density (**J**) is calculated by $e{n}_{e} ({{{{{{\bf{V}}}}}}}_{i}-{{{{{{\bf{V}}}}}}}_{e})$, where *e* is the charge of the electron, *n*_*e*_ is the electron density, **V**_*i*_ is ion bulk flow, and **V**_*e*_ is electron bulk flow. The energy dissipation in the electron frame is calculated by ${{{{{\bf{J}}}}}}\cdot ({{{{{\bf{E}}}}}}+{{{{{{\bf{V}}}}}}}_{e}\times {{{{{\bf{B}}}}}})$, where **J** is current density, **E** is the electric field, **V**_*e*_ is electron bulk flow, and **B** is the magnetic field. The phase space density (*f*) is calculated by

1 $$f=j/{{{{{{\bf{p}}}}}}}_{rel}^{2}$$

where *j* is differential flux, and ${{{{{{\bf{p}}}}}}}_{rel}$ is the relativistic momentum ($\gamma {m}_{e}{{{{{\bf{v}}}}}}$).

### Identification of current spikes

We manually identify the local maxima in $|{{{{{\bf{J}}}}}}| > 30\,{{{{{{\rm{nA}}}}}}/{{{{{\rm{m}}}}}}}^{2}$ as the current spikes. Two consecutive local maxima are considered independent spikes if the local minimum between them is less than the half-maximum of either peak. The duration between the two local minima is defined as the duration of the current spikes.

### Estimation of the induced electric field

According to Faraday’s law

2 $$\nabla \times {{{{{\bf{E}}}}}}=-\frac{\partial {{{{{\bf{B}}}}}}}{\partial {{{{{\boldsymbol{t}}}}}}}$$

and the conservation of magnetic flux

3 $${B}_{0}{{R}_{0}}^{2}={B}_{t}{({R}_{0}+V\delta {{{{{\boldsymbol{t}}}}}})}^{2}$$

we can get an estimate of the induced electric field caused by the expansion (or constriction) of the vortex structure,

4 $$|{{{{{\rm{E}}}}}}|=2{B}_{0}V$$

where *B*_0_ is the average magnitude of the magnetic field in the structure, *R*_0_ is the scale of the structure, and *V* is the expanding (or constricting) speed of the structure. An order of magnitude estimate was made for the induced electric field in the structure shown in Fig. 7. The average magnitude of the magnetic field (*B*_0_) was about 11 nT, and the expanding speed was represented by the mean electron bulk velocity (*V*_0_ ~ 2000 km/s). We estimated the induced electric field, *E* = 40 mV/m, which was comparable to the observed electric field (Fig. 7g).

## Supplementary information

Supplementary information

## Data Availability

All the MMS data used in this work are available at the MMS data center (https://lasp.colorado.edu/mms/sdc/public/about/browse-wrapper/), including magnetic field data (https://lasp.colorado.edu/mms/sdc/public/data/mms1/fgm/brst/l2/2017/05/28), electric field data (https://lasp.colorado.edu/mms/sdc/public/data/mms1/edp/brst/l2/dce/2017/05/28), particle data (https://lasp.colorado.edu/mms/sdc/public/data/mms1/fpi/brst/l2/des-moms/2017/05/28 for electrons and https://lasp.colorado.edu/mms/sdc/public/data/mms1/fpi/brst/l2/dis-moms/2017/05/28 for ions), energetic electron data (https://lasp.colorado.edu/mms/sdc/public/data/mms1/feeps/brst/l2/electron/2017/05/28), and magnetic field fluctuation data (https://lasp.colorado.edu/mms/sdc/public/data/mms1/scm/brst/l2/scb/2017/05/28). The AE (Auroral Electrojet) index used to estimate the substorm is available at the WDC for Geomagnetism (https://wdc.kugi.kyoto-u.ac.jp/ae_provisional/201705/index_20170528.html). The datasets generated during and/or analyzed during the current study are available from the corresponding author on reasonable request.

## Code availability

MMS data have been loaded, analyzed, and plotted using the SPEDAS software (Space Physics Environment Data Analysis Software). The SPEDAS software can be downloaded via the http://spedas.org/blog/ Downloads and Installation page.
