# Supplementary material for: Three-dimensional network of filamentary currents and super-thermal electrons during magnetotail magnetic reconnection
Source: Nat Commun. 2022 Jun 10;13:3241. doi: 10.1038/s41467-022-31025-9 (PMC9187682; doi:10.1038/s41467-022-31025-9)
Supplement: Supplementary file 1 — Supplementary information [file 41467_2022_31025_MOESM1_ESM.pdf]

Supplementary information for

Three-Dimensional Network of Filamentary Currents and Super-Thermal Electrons during Magnetotail Magnetic Reconnection

Xinmin Li[1,2,3], Rongsheng Wang[1,2,3*], Quanming Lu[1,2,3#], Christopher T. Russell[4], San Lu[1,2,3], Ian J. Cohen[5], R.E. Ergun[6], Shui Wang[1,2,3]

[1]CAS Key Laboratory of Geospace Environment, Department of Geophysics and Planetary Science, University of Science and Technology of China, Hefei 230026, China

[2]CAS Center for Excellence in Comparative Planetology, China

[3]Anhui Mengcheng Geophysics National Observation and Research Station, University of Science and Technology of China, Mengcheng 233500 Anhui, China.

[4]Earth Planetary and Space Sciences, University of California, Los Angeles, CA 90095, USA

[5]The Johns Hopkins University Applied Physics Laboratory, Laurel, MD, USA

[6]Department for Astrophysical and Planetary Sciences, University of Colorado, Boulder, CO, USA

**rswan@ustc.edu.cn; [#]qmlu@ustc.edu.cn*

Supplementary Information includes:

Supplementary Figs. 1-4

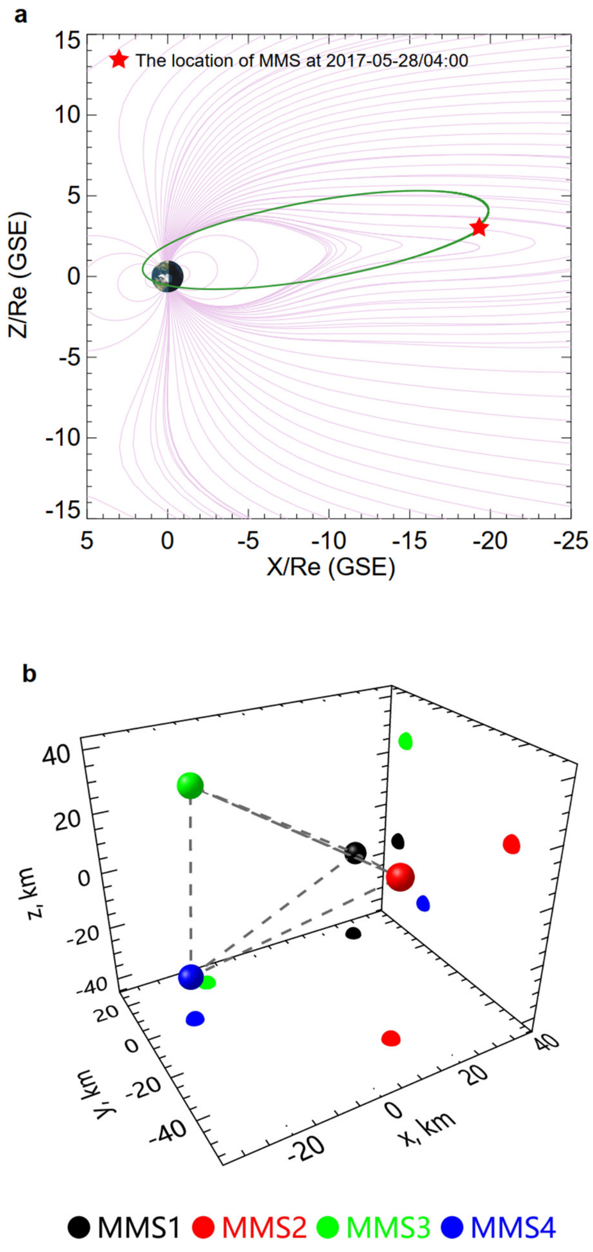

Supplementary Figure 1. Locations of MMS spacecraft during the magnetotail crossing. a, x-z plane projection of MMS orbit in GSE coordinates. MMS was at [-19.2, -11.3, 3.2] Re in Geocentric Solar Ecliptic (GSE) coordinates. **b**, MMS tetrahedral formation in GSE coordinates. The separations between the four spacecraft were minimal. The smallest separation was between MMS1 and MMS4, only 55.5km ~3.2 d_e (d_e is the electron inertial length).

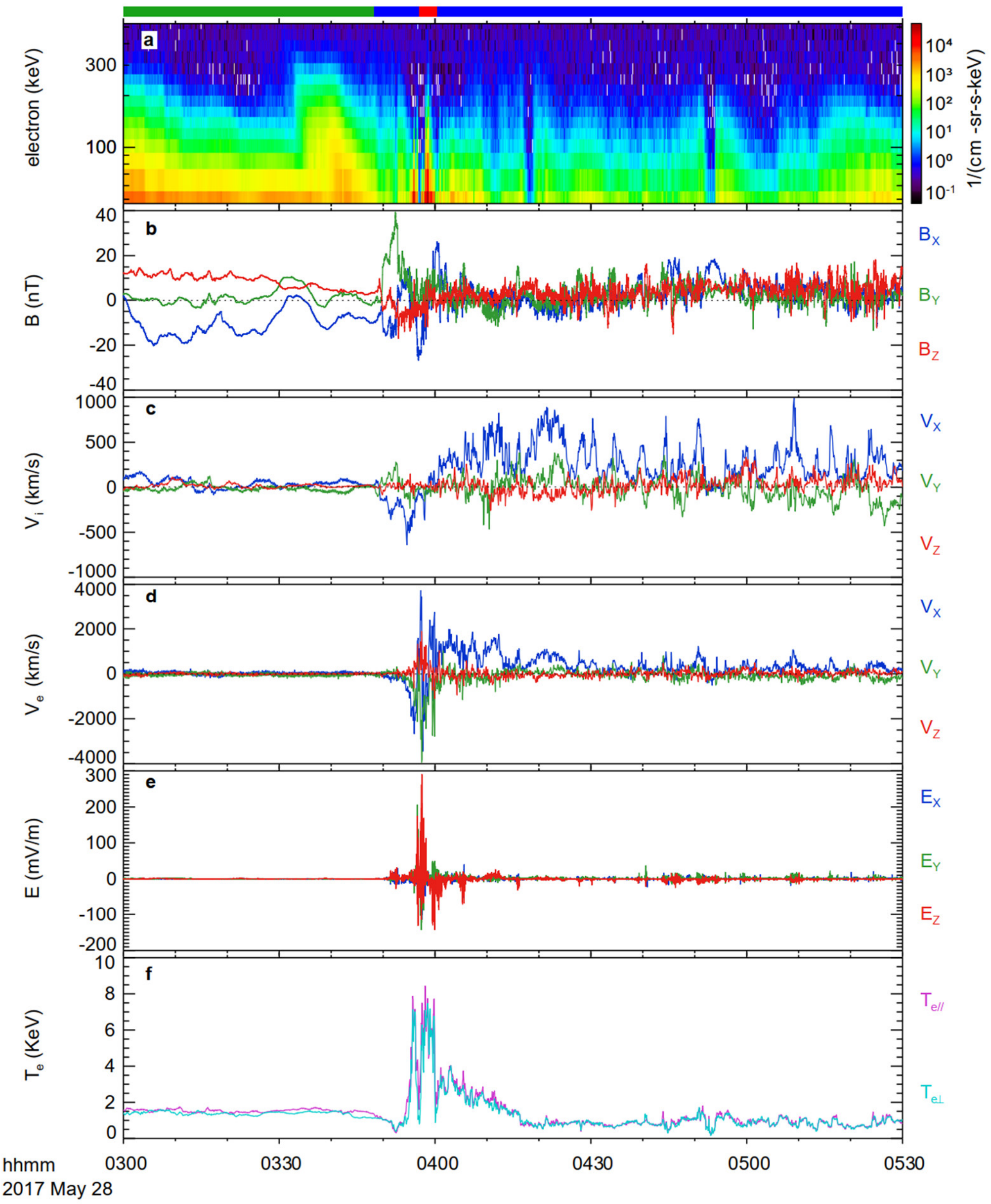

Supplementary Figure 2. MMS1 fast survey observations of this turbulent reconnection event. a, Energetic electron (47 – 500 keV) omnidirectional differential flux; **b**, Three components of the magnetic field; **c**, The ion bulk flows; **d**, The electron bulk flows; **e**, Three components of electron field; **f**, Parallel and perpendicular electron temperatures. The blue bars represent the outflow region of reconnection characterized by the intense ion bulk flow. The green bar represents

the inflow region characterized by the relatively quiet plasma flow, low and stable electron temperature, and a large magnetic field B_x. The red bar denoted the diffusion region investigated in the main text.

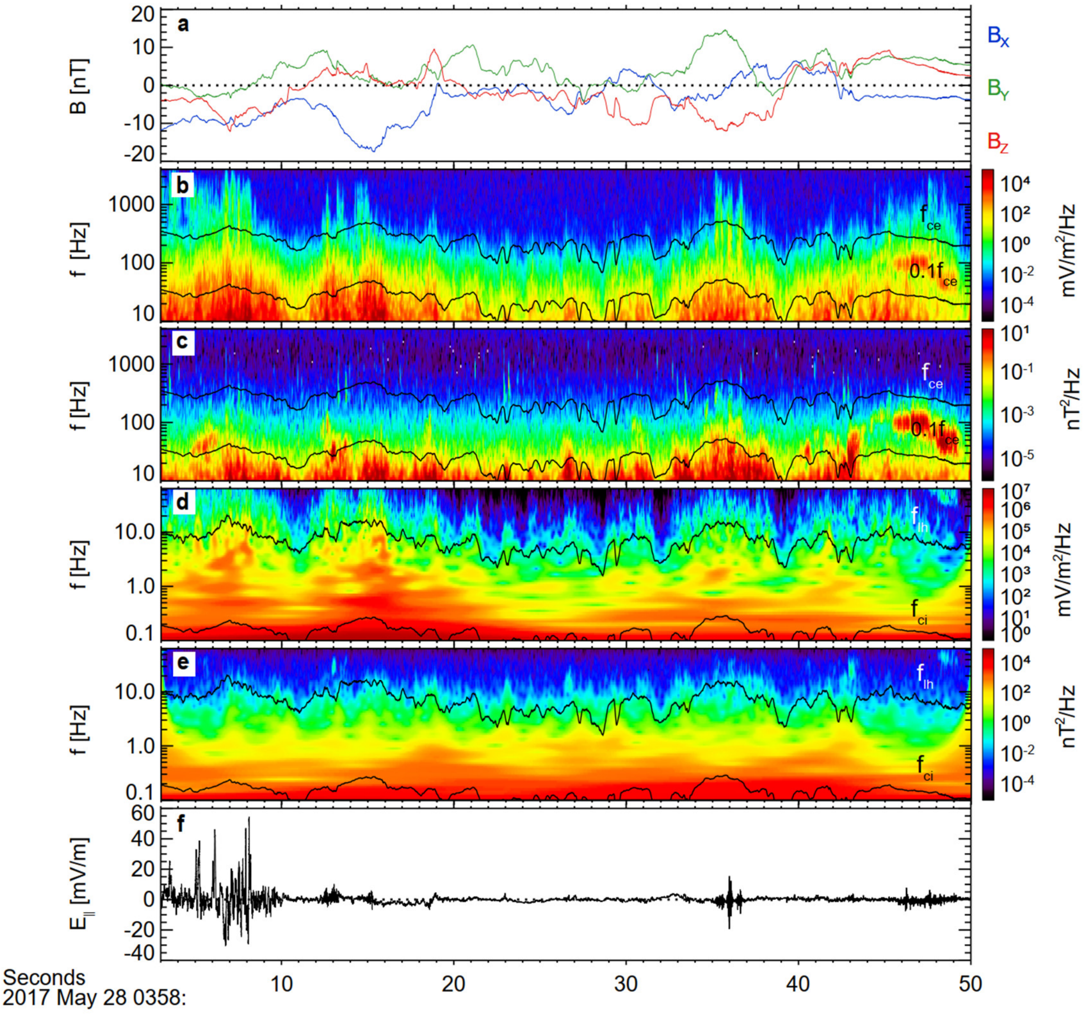

Supplementary Figure 3. Wave information around the X-line. a, magnetic field. **b–c**, high-frequency magnetic field and electric field power spectral densities according to the wavelet technique. Several enhancements of magnetic and electric field fluctuations were observed at $f = 0.1 - 1 f_{ce}$ (f_{ce} is the electron gyrofrequency), suggesting that these fluctuations were electromagnetic whistler-mode waves.

Moreover, many broadband fluctuations were observed above the f_{ce}, especially in the spectrogram of the electric field, indicating that there were a lot of high-frequency electrostatic and electromagnetic fluctuations. **d–e,** low-frequency magnetic field and electric field power spectral densities according to the wavelet technique. Both electric and magnetic fields had fluctuations between f_{ci} and f_{lh} (f_{ci} is the proton gyrofrequency and f_{lh} is the lower hybrid frequency), suggesting that the low-frequency waves also existed around the X-line. **f**, the parallel component of the electric field. The bipolar $E_{||}$ structures could correspond to the electrostatic solitary waves (electron holes).

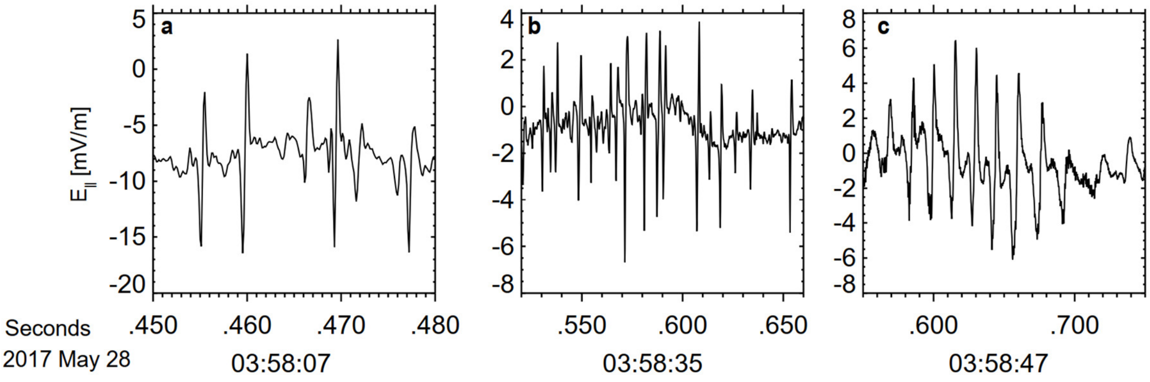

Supplementary Figure 4. Some examples of electron holes around the X-line. a-c, the parallel component of the electric field, corresponding to the electrostatic solitary waves (electron holes).
